# Identification of suitable reference genes for real-time qPCR in homocysteine-treated human umbilical vein endothelial cells

**DOI:** 10.1371/journal.pone.0210087

**Published:** 2018-12-31

**Authors:** Xia Zhu, Lujun Zhang, Yangxi Hu, Jianliang Zhang

**Affiliations:** 1 Department of Cardiology, Changhai Hospital, Naval Medical University, Shanghai, China; 2 Department of Cardiology, Urumqi Friendship Hospital, Urumqi, China; University of Naples Federico II, ITALY

## Abstract

The imbalance in homocysteine (Hcy) metabolism has been implicated in the pathogenesis of human diseases, including cardiovascular and neurodegenerative disorders. When attempting to identify gene expression profiles using quantitative real-time reverse transcription polymerase chain reaction (RT-qPCR), the selection of suitable reference genes is important. Here, the expression levels of 10 commonly used reference genes were assessed for normalization of RT-qPCR in Hcy-treated human umbilical vein endothelial cells (HUVECs) and control cells. The suitability of eight selected candidate genes was comparatively analyzed across the tested samples and separately ranked by four programs, geNorm, NormFinder, BestKeeper, and the ΔCt method. *Glyceraldehyde-3-phosphate dehydrogenase (GAPDH)* was the most stable gene in the final ranking using the RankAggreg package. Surprisingly, the *β-actin* (*ACTB*) levels decreased significantly in Hcy-treated HUVECs compared with control HUVECs (*P*<0.05), and further study indicated that Hcy suppressed the expression of *ACTB* by upregulating the miR-145-5p level in Hcy-treated HUVECs. Our data suggest that *GAPDH* can be used as a reliable reference gene, while *ACTB* cannot; normalization of gene expression in RT-qPCR experiments in Hcy-treated HUVECs. The data, which identifies a suitable reference gene in Hcy-treated HUVECs, will contribute to the design of an effective and accurate method for quantitation of gene expression.

## Introduction

Homocysteine (Hcy) is a nonessential sulfur -containing amino acid, a demethylation product generated during the metabolism of methionine [1, 2]. Hyperhomocysteinemia (HHcy) has been recognized as an independent risk factor for cardiovascular disease (CVD) [3]. The abnormally high level of Hcy in the circulating blood is associated with pathological changes such as atherosclerosis, nutritional deficiencies and malignancies [4]. The mechanisms underlying Hcy-induced pathogenesis includes cell apoptosis and proliferation, aggregation of platelets and increasing lipid peroxidation [5]. Hcy induces the apoptosis of human umbilical vein endothelial cells (HUVECs) by the physiological dysfunction of endoplasmic reticulum (ER) [6].

Gene expression analysis provides critical information for the underlying mechanism in an Hcy-induced model, and quantitative real-time reverse transcription polymerase chain reaction (RT-qPCR) is widely used to measure gene expression at the level of transcription. qPCR has emerged as a well-established and facile technique due to its fast, accurate and sensitive evaluation of mRNA expression in biological samples [7]. Suitable application of this method requires normalization using a reference gene to account for variation in the selection of starting material of RNA quality, cDNA synthesis efficiencies and differences between tissues and cell types [8]. The selection of suitable reference genes that show stable expression under special experimental conditions is critical for effective internal control of errors [9]. Several studies, however, have confirmed that some housekeeping genes are not consistently expressed under certain experimental conditions [8].

Micro RNAs (miRNAs) are short (20–25 nucleotides) non-coding RNAs that regulate gene expression by inhibiting translation or inducing degradation of target mRNA [10]. These target genes may include the widely used reference gene. *Glyceraldehyde-3-phosphate dehydrogenase* (*GAPDH*) and *β-actin* (*ACTB*) are direct targets of miR-644a and are not suitable as reference genes in miR-644a functional experiments [11]. High doses of homocysteine (Hcy) induce differential expression of 11 miRNAs by upregulating the level of dicer in cardiomyocytes [12]. A previous study reported that miR-145 is involved in regulating vascular smooth muscle cell (VSMC) phenotypes [13] in an Hcy-induced VSMC proliferation model. Whether the expressions of housekeeping gene are changed by miRNA regulation needs to be considered in Hcy-treated cells.

To date, no formal evaluation of suitable reference genes has been performed on Hcy-induced tissues or cell types. The aim of our study was to identify the stable reference gene for real-time qPCR in Hcy-treated HUVECs. A total of 10 frequently widely used reference genes (*HPRT*, *Rpl13A*, *18s rRNA*, *U6*, *YWHAZ*, *PPIA*, *GAPDH*, *ACTB*, *B2M* and *TPB*) were selected as candidate reference genes from previous reports [8, 14–15]. Their expressions were evaluated using RT-qPCR in Hcy-treated HUVECs and control HUVECs. To determine the appropriate reference genes, stable gene(s) were determined using four statistical algorithms: geNorm, NormFinder, BestKeeper and the ΔCt method. The RankAggreg package was used to identify the most stable reference genes according to consensus rankings. *GAPDH* was identified as the most reliable reference gene in Hcy-treated HUVECs. MiR-145-5p was induced by Hcy, which negatively regulated *ACTB*. Thus, *ACTB* was not suitable as a reference gene in Hcy-treated HUVECs. The identification of an appropriate reference gene will be valuable for the normalization of real-time qPCR data for gene expression studies in Hcy-treated HUVECs.

## Materials and methods

### Cell culture and treatment

HUVECs obtained from American Type Cell Collection (Manassas, VA, USA) were cultured in Dulbecco’s Modified Eagle Medium (DMEM; HyClone, Shanghai, China) containing 10% fetal bovine serum (Gibco, Logan, UT, USA), 1% glutamine (HyClone, Shanghai, China), 100 U/mL penicillin and 100 *μ*g/mL streptomycin (HyClone, Shanghai, China). The cells were grown in 6 well plates within a humidified incubator at 37°C in a 5% CO_2_ environment. Cells that reached 80% confluence were transfected with miR-145 inhibitor or the negative control inhibitor (RiboBio Co., LTD, Guangzhou, China) using Lipo2000 reagent (Invitrogen, Carlsbad, CA, USA) according to the manufacturer’s instructions. Briefly, a total of 200nM of the inhibitor (miR-145 inhibitor or the negative control inhibitor) and lipo2000 reagent (6μL) were independently diluted in 100μL Opti-MEM (Gibco, Shanghai, China) followed by incubation for 5min at room temperature. The two reagents were quickly mixed to form a transfection complex (total volume of approximately 200μL). Six hours after transfection, the culture medium was discarded and replaced by fresh medium, followed by the addition of homocysteine (Sigma-Aldrich, St Louis, MO, USA) with a final concentration 3 mM for 24 h. After treatment, the cells (n = 14 in each group) were collected for further experiments.

### RNA isolation and reverse transcription

RNA from the cells was extracted using TRIzol Reagent (Invitrogen, Carlsbad, CA, USA) following the manufacturer's instructions. Total RNAs were diluted with DNase/RNase-free water and stored at −80°C until use. RNA quality was analyzed basing on strict processes as indicated in the Minimum Information for Publication of Quantitative Real-Time PCR Experiments (MIQE) guidelines [16]. The concentration of RNA was identified by the optical density (*OD*) at 260 nm, and the purity of RNA was determined the 260 nm/280 nm ratio with expected values between 1.8 and 2.0 using the ND-1000 spectrophotometer (NanoDrop Technologies, Winooski, VT, USA). Total RNA integrity was evaluated by electrophoresis on 2% (w/v) agarose gels according to the 28S to 18S rRNA ratio. For the first strand cDNA synthesis, the extracted RNAs were reverse transcribed in a total volume of 20 μL using a FastQuant RT Kit (cat #KR106; TianGen, Bejing, China) according to the manufacturer's instructions. To remove contaminating DNA, the first reaction mixture consisted of 2 μL 5× gDNA Buffer and 1000 ng RNA template in a total volume of 10 μL, and followed by incubation at 42°C for 3 min. The cDNA reaction mixture containing 2 μL 10× Fast RT Buffer, 1 μL RT Enzyme Mix, 2 μL FQ-RT Primer Mix, 2 μL Stem-Loop RT Primer Mix (0.5 μL of equal volume for miR-145-5p, miR-1-3p, miR-124-3p, and miR-205-5p) and 3 μL RNase-free H_2_O, were added to the last reaction mixture to a total volume of 20 μL. Reverse transcription was performed on a Gene Amp PCR System 9700 (Applied Biosystems, Foster City, CA, USA) at 42°C for 15 min and 95°C for 3 min. All reactions were performed in triplicate, including the no-template control. The produced cDNA were stored immediately at −20°C for later use.

### Real-time PCR

The qPCR was run using the ABI Prism7900 system (Applied Biosystems, Foster City, CA, USA) using the SYBR Green Real time PCR Master Mix (TOYOBO, Tokyo, Japan) according to the manufacturer’s protocol and the MIQE guidelines (http://www.rdml.org/miqe). Each 20 μL reaction consisted of 10 μL of SYBR Green Real time PCR Master Mix, 0.4 μL of Forward Primer (10 μM), 0.4μL of Reverse Primer (10 μM), 2 μL of cDNA and 7.2 μL of RNase-free H_2_O. The reaction of qPCR was performed using the program: 95°C for 30 s, followed by 40 cycles of 95°C for 5 s, 60°C for 10 s and 72°C for 15 s. The qPCR products were verified with a melting curve at a range of 55–99°C. All samples were measured in triplicate to ensure reproducibility.

### Western blot assay

Cells were collected and then total protein was extracted using a Protein Extraction Kit (KGP9100; Key Gene, Nanjing, China) according to the manufacturer’s protocol. The concentration of the extracted protein was tested by a BCA protein assay kit (Beyotime, Bejing, China) followed by an immunoblotting assay. Briefly, equal 20 μg aliquots of total protein were separated by 12% sodium dodecyl sulfate–polyacrylamide gel electrophoresis at 90V for 15 min followed 120V for approximately 50 min. The proteins were transferred to a polyvinylidene fluoride membrane with a constant current at 200 mA for 60 min. The membrane was blocked with 5% nonfat milk for 2 h at room temperature, followed by incubation with primary antibodies (1: 5000 dilution) at 4°C for 12–16 h. After washing with Tris-buffered saline containing Tween 20 (TBST), the membranes were incubated with horseradish peroxidase (HRP)-labeled secondary antibodies (1: 5000 dilution) for 2 h at room temperature. After extensive washing with TBST, the signals of the bands were visualized using Luminata Crescendo Western HRP Substrate (Millipore, Billerica, MA, USA). The antibodies including those to ACTB and GAPDH, and secondary antibodies were purchased from Abcam (Cambridge, MA, USA).

### Statistical analysis

Four common algorithms (geNorm, NormFinder, BestKeeper, and the ΔCt method) were performed to individually examine the expression stability of the eight candidate reference genes. GeNorm, NormFinder, and BestKeeper are commonly applied software programs based on Microsoft Excel. GeNorm and NormFinder require the relative expression value (the RQ value) for each sample. RQ = E ^ (Ct _*(mean)*_*—*Ct _*(sample)*_), where E = efficiency of the primers for each gene and Ct _*(mean)*_ = the average of all the Ct values of samples. Ct values are used in BestKeeper and the comparative ΔCt method. Based on a pairwise comparison approach, the geNorm program [17] determines the best reference gene by calculating its average pairwise variation relative to all other tested samples (M-value). The gene with the lowest M-value is the most stably expressed, while the gene with the highest M-value is the least stably expressed. The M-values was set below the default limit of 1.5 to evaluate gene stability [17]. By grouping samples, NormFinder [18] uses a model-based variance estimation algorithm based on intra- and inter-group variations to estimate the single best stable reference gene. The BestKeeper [19] applet evaluates the gene expression variation for individual reference genes to determine the optimal reference genes according to the coefficient of variation (CV) [20]. The candidate gene with the least SD is considered to be the most stable gene. The ΔCt method uses the mean SD of all candidate genes’ Ct values to identify the most stable genes [21]. This statistical algorithm ranks the stability of reference genes by comparing the Ct values between two genes in all tested samples, considering the gene with the least SD as the best reference gene. Finally, the RankAggreg package of the R project was applied to establish a consensus rank basing on the result from the four methods above according to previous study.

Statistical analysis were performed with SPSS 19.0 statistical software (SPSS Inc., Chicago, IL, USA). Data were presented as mean ± standard deviation (Mean ± SD), significant differences were analyzed between the two groups using the Student’s t-test and the Wilcoxon–Mann–Whitney test. The remains were determined with one way-analysis of variance (ANOVA). A P-value < 0.05 indicated statistical significance.

## Results

### Evaluation of expression stability

The expressions of 10 genes were evaluated in all tested samples by RT-qPCR and were selected from commonly used reference genes. Their full names, accession numbers, primer sequences and the amplicon length of the products are provided in Table 1. For each primer pair, standard curves were obtained to estimate the stability of the gene detection, as in a previous report [22]. The linear correlation coefficients (*R*^2^), an indicator of fit for the standard curves, had expected values between 0.993 and 1.000, and the amplification efficiencies (*E* value) ranged from 95% to 107% in all genes. Each gene showed an individual PCR product with a single homogenous melting peak after melting curve analysis. Only eight genes (*Rpl13A*, *18s rRNA*, *U6*, *YWHAZ*, *PPIA*, *GAPDH*, *B2M* and *TPB*) could be used as candidate reference genes according the criteria, as in a previous report [22]. The exceptions were *HPRT* and *ACTB*, which were expressed at different levels between the two groups of cells.

**Table 1:** Primers for candidate reference genes.

Symbol	Accession	Name	Primer Sequences (5'-3')		Am (bp)
HPRT	NM_000194	Hypoxanthine phosphoribosyl-transferase	F: CCTGGCGTCGTGATTAGTGAT	R:AGACGTTCAGTCCTGTCCATAA	131
Rpl13A	NM_012423	Ribosomal protein L13A	F:GCCCTACGACAAGAAAAAGCG	R:TACTTCCAGCCAACCTCGTGA	117
18s rRNA	NR_046235.1	18s subunit ribosomal RNA	F:AGAAACGGCTACCACATCCA	R:CACCAGACTTGCCCTCCA	169
U6	NR_004394.1	U6 small nuclear 1	F:CTCGCTTCGGCAGCACA	R:AACGCTTCACGAATTTGCGT	94
YWHAZ	NM_001135702	Tyrosine 3-monooxygenase/tryptophan 5-monooxygenase activation protein, zetapolypeptide	F:CCTGCATGAAGTCTGTAACTGAG	R:GACCTACGGGCTCCTACAACA	100
PPIA	NM_021130	peptidylprolyl isomerase A	F: ATGTGTCAGGGTGGTGACTTC	R:GCCATCCAACCACTCAGTCTT	118
GAPDH	NM_001256799	Glyceraldehyde-3-phosphate dehydrogenase	F:ACAACTTTGGTATCGTGGAAGG	R:GCCATCACGCCACAGTTTC	101
ACTB	NM_001101.3	Beta-actin	F:CGACAGGATGCAGAAGGAG	R:ACATCTGCTGGAAGGTGGA	137
B2M	NM_004048	Beta-2 microglobulin	F:TCCAGAAACTAATGGCAGATCCC	R:AATTCCCTACGCTTTGGGTTTT	163
TBP	NM_003194	TATA box binding protein	F:TGCACAGGAGCCAAGAGTGAA	R:CACATCACAGCTCCCCACCA	132

F: Forward primer; R: Reverse primer; Am: amplicon size; bp: numer of base pairs.

### Analysis of reference gene ranking

**Fig 1:**
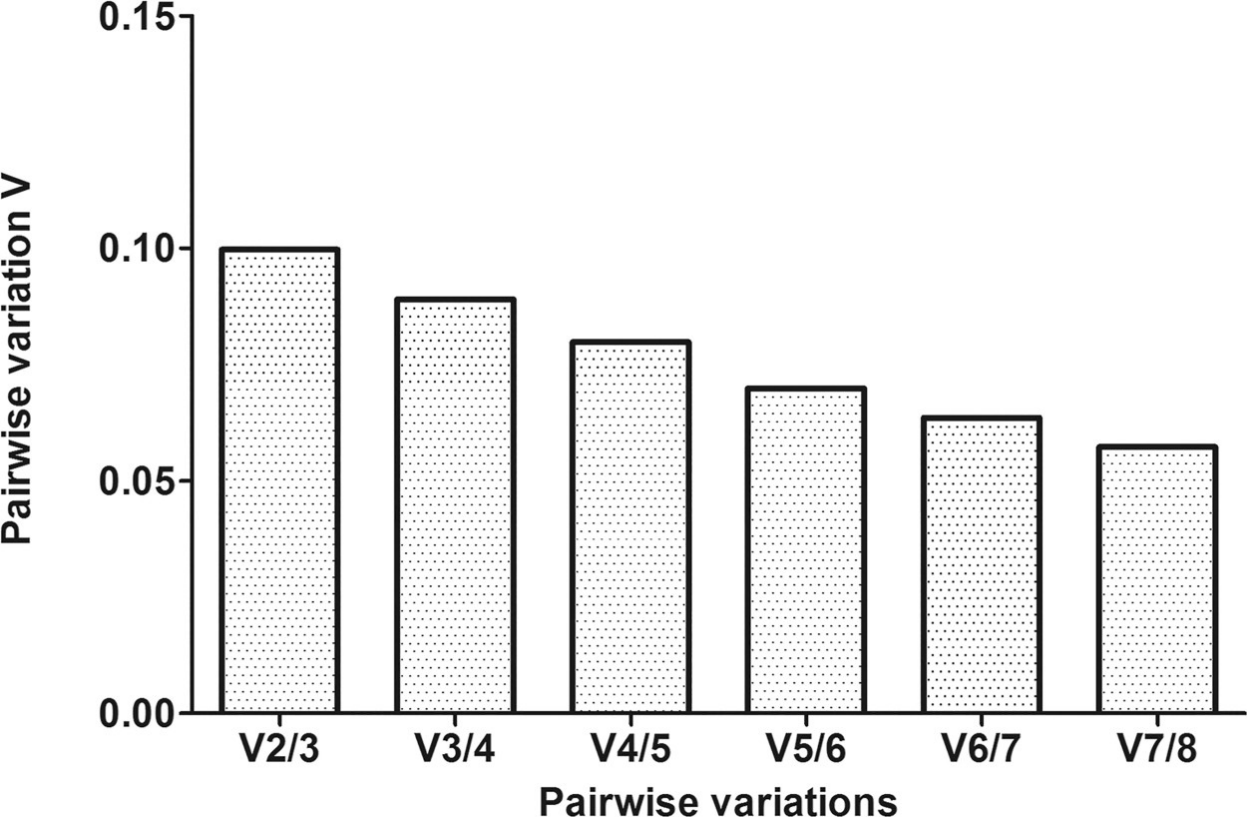
Evaluation of reference genes using geNorm across all tested samples. Determination of the optimal number of reference genes for normalization relied on the pairwise variation (*Vn/n + 1*) analysis. Every bar represents a change in normalization accuracy with the stepwise addition more of reference genes. The figure indicates that there is no need to include more than two genes into the normalization factor.

As shown in Table 2, geNorm ranked *U6* and *TPB* as the most stable pairwise combination of reference genes, followed by *GAPDH*. Next, the pairwise variation value (*V/NF* value), a criterion for the optimum number of reference genes, was analyzed according to a previous report [8]. The first *V*-value (*V2/3*) was < 0.15, suggesting that only the two genes are sufficient for a reliable normalization (Fig 1).

**Table 2:** Ranking order of the candidate reference genes by four programs: geNorm, NormFinder, BestKeeper and ΔCt.

Rank	geNorm		NormFinder		BestKeeper		ΔCt	
	Gene	Stability (M)	Gene	variability	Gene	CV (%Ct)	Gene	Mean SD
1	U6/TPB	0.172	GAPDH	0.066	GAPDH	0.44	GAPDH	0.322
2			U6	0.132	TPB	0.95	B2M	0.352
3	GAPDH	0.249	TPB	0.145	U6	1.08	U6	0.359
4	B2M	0.275	B2M	0.147	B2M	1.3	TPB	0.364
5	Rpl13A	0.291	ppIA	0.199	ppIA	1.59	Rpl13	0.393
6	ppIA	0.312	Rpl13A	0.207	Rpl13A	1.61	YWHAZ	0.408
7	YWHAZ	0.357	YWHAZ	0.294	YWHAZ	1.74	ppIA	0.415
8	18s rRNA	0.409	18s rRNA	0.347	18s rRNA	2.77	18s rRNA	0.595

M: the mean expression stability values; CV: the coefficient of variance; Mean SD: mean standard deviation.

Table 2 illustrates that NormFinder ranked *GAPDH* as the most stable gene with an M-value of 0.066, followed by U6 with an M-value of 0.132. With BestKeeper, *GAPDH* was ranked as the most stable gene with a CV (%Ct) of 0.44, followed by TPB with a CV of 0. 0.95. The ΔCt method indicated that *GAPDH* was the most stable reference gene with a mean standard deviation of 0.322, followed by *B2M* with a mean standard deviation of 0.352.The final ranking of reference genes was performed using the RankAggreg package that integrated the results obtained from geNorm, NormFinder, BestKeeper, and the ΔCt method (Table 3). The final rankings revealed that *GAPDH* was the most suitable reference genes (Table 3).

**Table 3:** Final rankings of candidate reference genes.

Rank	geNorm	NormFinder	BestKeeper	ΔCt	Final Fank
1	U6/TPB	GAPDH	GAPDH	GAPDH	GAPDH
2		U6	TPB	B2M	U6
3	GAPDH	TPB	U6	U6	TBP
4	B2M	B2M	B2M	TPB	B2M
5	Rpl13A	ppIA	ppIA	Rpl13	Rpl13A
6	ppIA	Rpl13A	Rpl13A	YWHAZ	ppIA
7	YWHAZ	YWHAZ	YWHAZ	ppIA	YWHAZ
8	18s rRNA	18s rRNA	18s rRNA	18s rRNA	18s rRNA

geNorm, NormFinder, BestKeeper and ΔCt method were combined to establish a consensus rank of the genes by RankAggreg package.

### MiR‑145-5p mediates the effect of Hcy on *ACTB* expression

#### Influence of Hcy on the expression of miR‑145-5p in HUVECs

**Fig 2:**
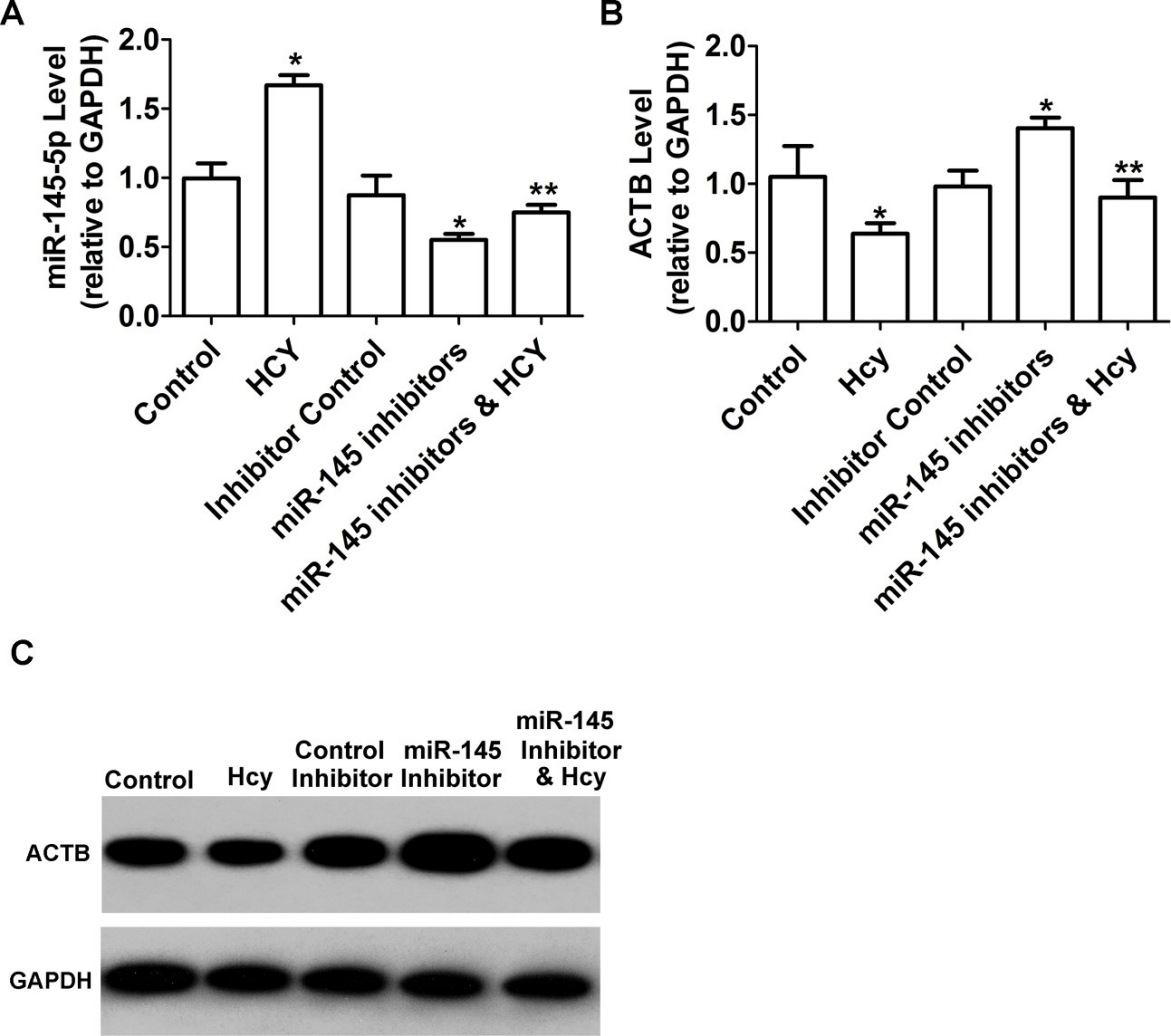
Comparisons of mir-145-5p and *ACTB* levels in the five groups. (A) Expressionof miR-145-5p were detected by RT-qPCR; (B-C) The mRNA and protein levels of *ACTB* was detected by RT-qPCR and western blot, respectively. * *P*<0.05 compared with control group; ** *P*<0.05 compared with miR-145 inhibitors group.

**Fig 3:**
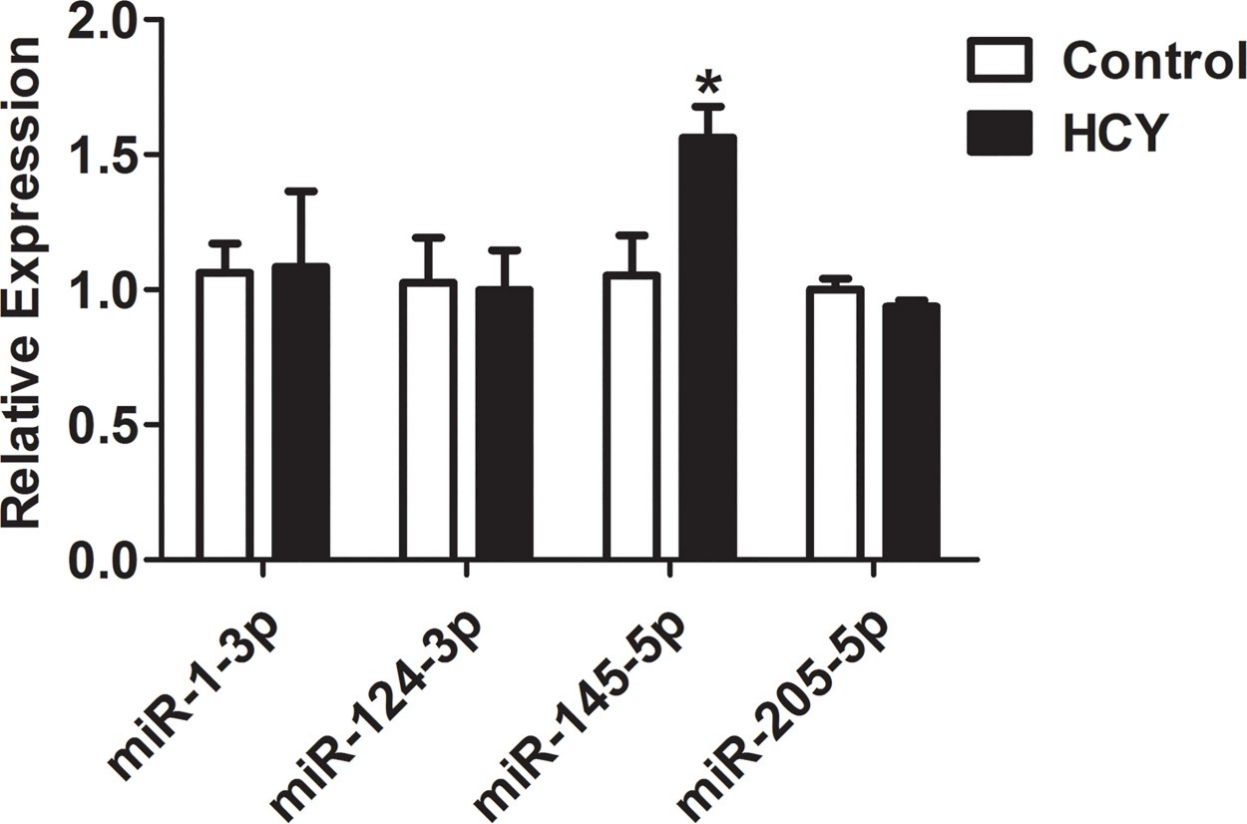
qRT-PCR analysis of miR-1-3p, miR-124-3p, miR-145-5p and miR-205-5p in Hcy-treated HUVECs. Only the miR-145-5p level increased significantly in the Hcy group compared with the control group. * *P*<0.05 when compared with control group.

*ACTB* is widely used as a reference gene in qPCR analysis [23–25]. However, in this study, the expression of *ACTB* was significantly down-regulated with a 0.63-fold change in Hcy-treated HUVECs (Fig 2B). To date, only four potential mature miRNAs (miR-1-3p, miR-124-3p, miR-145-5p and miR-205-5p) have been predicted to bind the 3’ untranslated region (UTR) of *ACTB* mRNA in the publicly available miRBase database (miRBase 21.0, http://www.mirbase.org). Retro-transcription primers and qPCR primers for these target miRNAs were designed (Table 4) to quantify their expression. When compared to the control group, the levels of three miRNAs (miR-1-3p, miR-124-3p, and miR-205-5p) showed no significant differences, but miR-145-5p level was significantly higher in Hcy-treated group (Fig 3).

**Table 4:** Primers for target miRNAs.

Symbol	Accession	Primer Sequences (5'-3')	Am (bp)
miR-145-5p	MIMAT0000437	RT:TGGAGCGACCGTGTCGTGGAGTCGGCTAATGGTCGCTCCAAGGGAT	77
		F:GACACTCCAGCAGCGGTCCAGTTTTCCCAGGAA	
		R:ATAGAGCGGTGTCGTGGAGTCGGCTAATGGTC	
miR-1-3p	MIMAT0000416	RT:TGGAGCGACCGTGTCGTGGAGTCGGCTAATGGTCGCTCCAATACAT	75
		F: GACACTCCAGCAGCGTGGAATGTAAAGAAGT	
		R:ATAGAGCGGTGTCGTGGAGTCGGCTAATGGTC	
miR-124-3p	MIMAT0000422	RT:TGGAGCGACCGTGTCGTGGAGTCGGCTAATGGTCGCTCCAGGCATT	74
		F:GACACTCCAGCAGCGTAAGGCACGCGGTGA	
		R:ATAGAGCGGTGTCGTGGAGTCGGCTAATGGTC	
miR-205-5p	MIMAT0000266	RT:TGGAGCGACCGTGTCGTGGAGTCGGCTAATGGTCGCTCCACAGACT	76
		F:GACACTCCAGCAGCGTCCTTCATTCCACCGGA	
		R:ATAGAGCGGTGTCGTGGAGTCGGCTAATGGTC	

RT: retro-transcription primer; F: Forward primer; R: Reverse primer; Am: amplicon size; bp: numer of base pairs.

#### MiR‑145-5p suppressed *ACTB* expression induced by Hcy

According to the levels of miR-145-5p and *ACTB* detected in the two groups, the Hcy-treated group had higher miR-145-5p expression than the control group, while the miR-145 inhibitor and Hcy group displayed lower miR-145-5p expression than the miR-145 inhibitor group (Fig 2A). When compared with inhibitor control group, the *ACTB* level was significantly increased by the miR-145 inhibitor. After treatment with Hcy, *ACTB* expression was significantly down-regulated in the miR-145 inhibitor and Hcy group compared with the miR-145 inhibitor group (Fig 2B). Its protein level also decreased significantly (Fig 2C).

## Discussion

RT-qPCR has been widely used to measure gene expressions in biological samples because of its speed, accuracy and sensitivity [26]. Reliable quantification requires appropriate normalization. However, no single reference gene is universally suitable for all purposes under different experimental conditions. For instance, miR-644a can target the 3’ untranslated region of *ACTB* and *GAPDH* mRNAs, indicating that *ACTB* and *GAPDH* are not suitable as reference genes in miR-644a functional studies [11]. To avoid misleading results, evaluating the expression stabilities of candidate reference genes under specified condition is an important prerequisite to normalizing the expression of other genes to the reference gene [27]. To our knowledge, the present study is the first detailed comparison of different normalization approaches for reference gene selection in Hcy-treated HUVECs. We analyzed the expression stabilities of ten commonly used candidate reference genes (*HPRT*, *ACTB*, *Rpl13A*, *18s rRNA*, *U6*, *YWHAZ*, *PPIA*, *GAPDH*, *B2M* and *TPB*). During the RT-qPCR validation stage, significant changes both in *HPRT* and *ACTB* levels were evident under the experimental condition of Hcy-treated HUVECs. Thus, *HPRT* and *ACTB* were omitted as candidate reference genes in the following steps. The remaining eight candidate reference genes were analyzed with four public algorithms (geNorm, NormFinder, BestKeeper, and the ΔCt method). geNorm identified *U6* and *TPB* as the most stable genes. The analysis of the NormFinder, BestKeeper, and ΔCt method indicated that *GAPDH* was the most stable reference genes, which was different than the order proposed by geNorm.

The different programs used to analyze the stability of candidate reference gene expressions did not produce the same results due to their different mathematical approaches [19]. As reported previously discrepancies in gene ranking occur using different programs [9]. Thus, in our study, the RankAggreg software package was performed to establish a consensus rank of these genes. The final rank was: *GAPDH* > *U6* > *TBP* > *B2M* > *Rpl13A* > *ppIA* > *YWHAZ* > *18s rRNA*. This data enriches the application of *GAPDH*, which was reliably expressed and is a widely used traditional reference gene in qPCR analysis [28, 29].

However, the data showed the *ACTB* levels were significantly lower in Hcy-treated cells. MiR-145-5p, one of the four miRNAs predicted to be the potential targeting *ACTB*, was up-regulated in Hcy-treated cells. Early research on miR-145 mainly focused on its association with the occurrence and development of cancer [30, 31]. Subsequent studies have demonstrated that miR-145 contributes to many cardiovascular diseases, including atherosclerosis [30, 32], pulmonary arterial hypertension [33], and acute myocardial infarction [34]. Overexpression of miR-145 via miR-145 mimics significantly inhibits VSMC proliferation induced by Hcy [13]. Clinical research has linked the level of circulating miR-145 to Hcy [35]. We also found the *ACTB* was down-regulated in Hcy-treated cells. The *ACTB* mRNA, which contains 599 nucleotides at 3′ UTR, is predicted to be a potential target of miR-145-5p by the Pictar, miRGen, miRnada, and TargetScan bioinformatics software programs [36]. To further confirm the relationship between miR-145 and *ACTB*, their expressions were examined in Hcy-treated cells transfected with the miR-145 inhibitor. As shown in Fig 2, when miR-145 expression was down-regulated by the miR-145 inhibitor, the effect of high *ACTB* level was blocked in Hcy-treated HUVECs. A previous study reported that a few housekeeping genes should be chosen carefully and cannot be used as reference genes for gene expression analysis under some conditions [37]. It was reported that miR-644a targets both *ACTB* and *GAPDH* by binding their mRNA 3ˈUTRs, suggesting that *ACTB* and *GAPDH* are not suitable as reference genes in miR-644a functional studies [11]. The similar results reflect the choice of *ACTB* as the reference gene in miR-145 functional experiments [38].Thus, it is possible that the role of Hcy in decreasing the *ACTB* level is mediated by miR-145-5p. Likewise, *Lox* mRNA expression was reportedly elevated 1.88±0.18-fold when normalized to *GAPDH*, and was elevated 2.36±0.16-fold when normalized to *ACTB* in HHcy mouse heart [39]. The higher expression of *Lox* mRNA may be due to its reference gene downregulation when chose *ACTB*. We chose *GAPDH* as a reliable reference gene just in Hcy-treated HUVECs at a high concentration of 3 mM. Moreover, as the effect of Hcy is cell- and tissue-specific, choosing *GAPDH*, but not *ACTB*, as the stable reference gene requires further verification in other biological systems. Nevertheless, our data support using *GAPDH*, not *ACTB*, as the reference gene for normalization of gene expression in RT-qPCR assays in Hcy-induced HUVECs.

However, further research on the role of miR-145 in targeting *ACTB* is necessary to verify this conclusion. We also did not focus on the various Hcy metabolites, such as homocysteine thiolactone and protein homocysteinylation. The expressions of 110 genes were significantly altered in response to Hcy-thiolactone treatment and 30 mRNAs were altered in response to N-Hcy-protein [40]. The metabolic conversion of homocysteine thiolactone and protein homocysteinylation in human endothelial cells were linked to Hcy-induced damage and vascular diseases, such as atherosclerosis [41].

## Conclusions

Our study shows that *GAPDH* is the most stable reference gene in Hcy–treated HUVECs. Hcy increased miR-145-5p levels, but decreased the *ACTB* levels. When the miR-145-5p level was down-regulated by the miR-145 inhibitor, the effect of Hcy on *ACTB* expression was reversed. The findings suggested *ACTB* may not be a suitable reference gene in miR-145-5p functional experiments.

## Supporting information

S1 Fig The full uncropped and un-altered blot image of ACTB. (TIF)

S2 Fig The full uncropped and un-altered blot image of GAPDH. (TIF)
