# Supplementary figures and images for: Identification of suitable reference genes for real-time qPCR in homocysteine-treated human umbilical vein endothelial cells
Source: PLoS One. 2018 Dec 31;13(12):e0210087. doi: 10.1371/journal.pone.0210087 (PMC6312244; doi:10.1371/journal.pone.0210087)

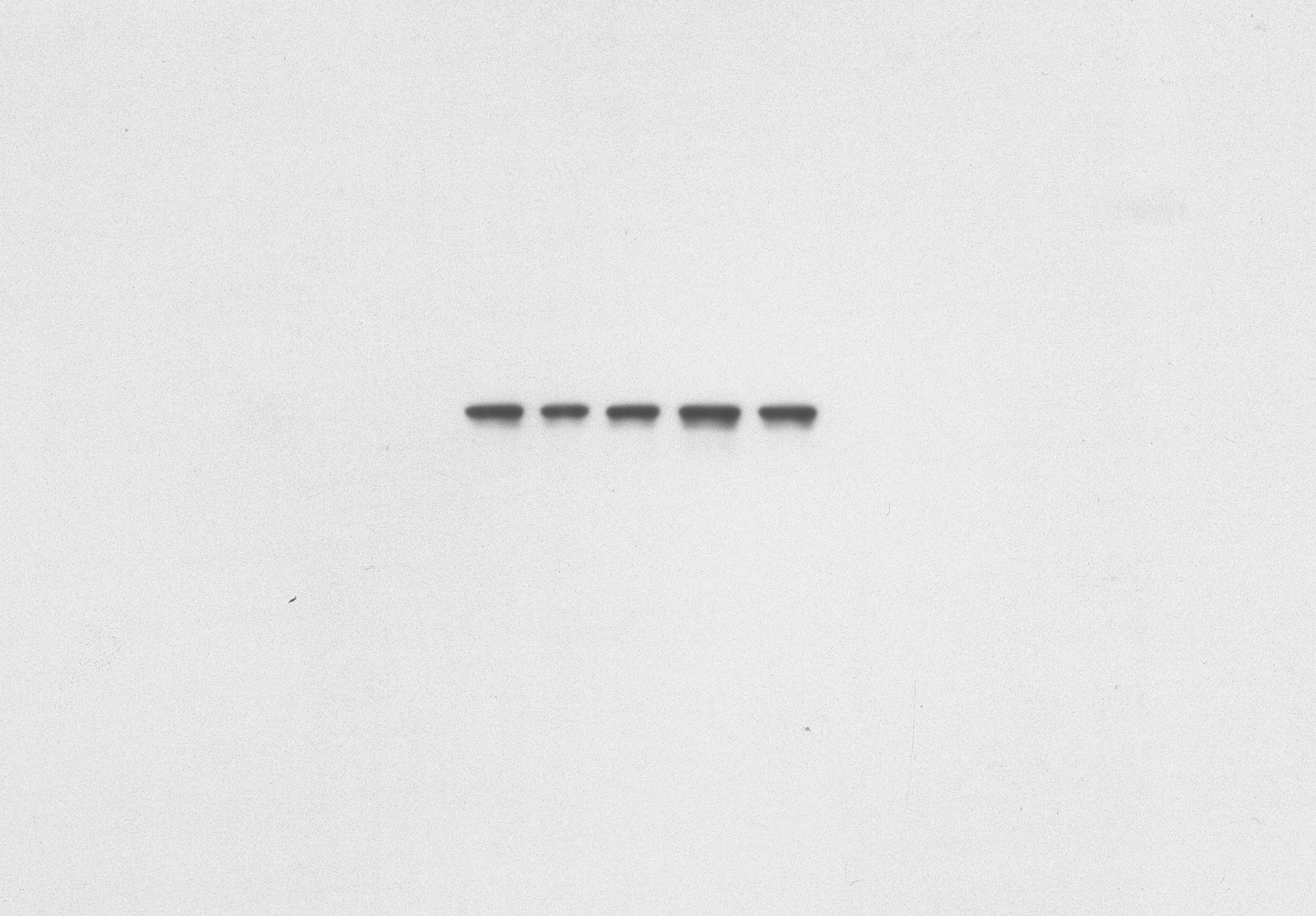

Supplement: S1 Fig — (TIF) [file pone.0210087.s001.tif]

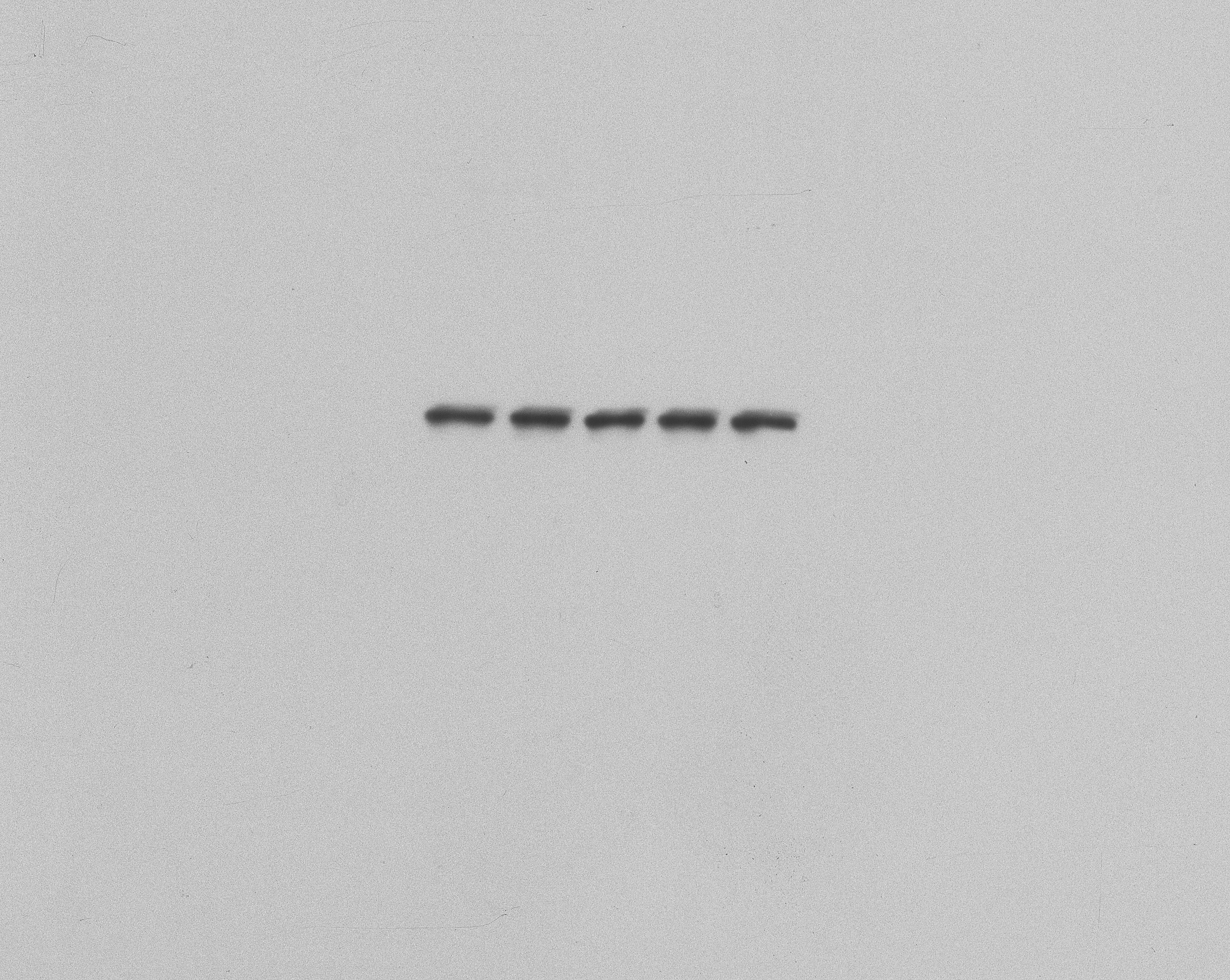

Supplement: S2 Fig — (TIF) [file pone.0210087.s002.tif]
